# Long-Term Results of Intensity Modulated Radiotherapy (IMRT) with Helical Tomotherapy in Non-Metastatic Breast Cancer Patients: Final Analysis

**DOI:** 10.3390/cancers17030544

**Published:** 2025-02-06

**Authors:** Pierre Loap, Abdelkarim Uakkas, Sofiane Allali, Jihane Bouziane, Alain Fourquet, Youlia Kirova

**Affiliations:** 1 Institut Curie, 25 rue d’Ulm, 75005 Paris, France; pierre.loap@curie.fr (P.L.); abdelkarim.uakkas@curie.fr (A.U.); sofiane.allali@curie.fr (S.A.); jihane.bouziane@curie.fr (J.B.);; 2 University of Versailles St Quentin, 78 Yvelines, 78000 Versailles, France

**Keywords:** breast cancer, intensity modulated radiotherapy, helicoïdal tomotherapy, efficacy, toxicity

## Abstract

**Background:** Intensity modulated radiotherapy with helical tomotherapy (IMRT-HT) is used in the breast cancer (BC) treatment for years now to obtain homogeneous dose distribution in the treated volumes and reduce the doses to organs at risk. The purpose of this study was to evaluate our experience in terms of local control, overall survival, progression free survival and adverse events in BC patients treated with IMRT-HT with long term follow-up. **Methods:** This study is a retrospective data analysis of patients irradiated with IMRT-HT. Overall survival (OS) and progression free survival (PFS) curves were plotted with Kaplan-Meier method. We also analyzed the OS and PFS data by molecular subgroups of the population. Long-term toxicities including skin, cardiac and pulmonary complications were also evaluated. Multivariant logistic regression analysis was performed to determine the independent predictors of the side effects. **Results:** Between 2009 and 2015, a total of 194 breasts in 179 women with nonmetastatic breast cancer were treated. Most of the tumors were grade III and N+. With a median follow-up of 10 years, we observed 9 local recurrences, 2 loco-regional recurrences, and 29 patients experienced metastatic disease. Only 18 patients are dear, of them 7 cases with breast cancer death. At 10 years, the Local recurrence free survival was 95.3% [95%CI: 92.1–98.5], the loco-regional relapse free survival was 94.5% [91.1–98.1]. The metastases free survival was 82.9% [76.9–89.3]. The progression free survival was 79.9 [73.6–86.7]. The cancer specific survival was 94.3%, and the overall survival 88% [82.8–93.5]. At long term, there were no cardiac, lung, thyroid, digestive radio induced toxicities. A small number of patients experienced grade I or II fibrosis. **Conclusions:** IMRT-HT could be safely used for adjuvant breast cancer irradiation in patients with complex anatomy. IMRT-HT provides favourable long-term prognosis, while late toxicity is acceptable.

## Simple Summary

Intensity modulated radiotherapy with helical tomotherapy (IMRT-HT) is used in the breast cancer (BC) treatment for years now to obtain homogeneous dose distribution in the treated volumes and reduce the doses to organs at risk. The present study has shown for breast cancer patients treated with IMRT-HT with long term follow-up that IMRT-HT could be safely used for adjuvant breast cancer irradiation in patients with complex anatomy, mostly N+ aggressive disease.

## 1. Introduction

The breast cancer is the first cancer as incidence and one of the first causes of mortality in the women all over the word [1]. The post-operative radiation therapy is a part of the multidisciplinary management of breast cancer after mastectomy or after breast conserving surgery with improvement of local and regional control, as well as the improvement of disease-free survival (DFS), and overall survival (OS) [2]. At the same time, the radiation therapy could be related to early and late side effects as fibrosis, lung and cardiac toxicity and extremely rare radiation induced sarcomas [3]. Lung and heart toxicities have been reported with the old techniques of radiation therapy, related to the dose exposure, but also to the heterogeneous irradiation [4,5].

Alternative techniques as treatment in lateral or prone position, as well as the deep inspiration breath hold (DIBH) have been developed to reduce the doses to heart and lung as main organs at risk (OAR) [6,7,8,9]. These techniques are not available in all departments of radiation oncology, therefore their use is limited. Other technique related to reduction of doses to organ-at-risk and improvement of target volume coverage is the intensity modulated radiotherapy (IMRT) in comparison with the classic 3D conformal radiation therapy [10,11]. Because the homogeneity and the reduction of doses to main organs at risk, the intensity modulated radiotherapy can be associated with reduced toxicity [12,13].

Previously we have studied and shown that the use of intensity modulated radiation therapy with Tomo therapy is extremely useful in population of patients treated for breast cancer with complex volumes of irradiation as bilateral cancers with or without lymph nodes, pectus excavatum, impossibility to use the deep inspiration breath hold [11,14,15]. The purpose of this study is initially scheduled final analysis at 10 years follow up of prospectively registered consecutive breast cancer patients, treated with intensity modulated radiotherapy (IMRT) by helical tomo therapy (HT) in our institution.

## 2. Materials and Methods

This retrospective study of prospectively registered consecutive breast cancer patients, treated with intensity modulated radiotherapy (IMRT) by helical tomotherapy (HT) was conducted in our Department of Radiation Oncology of the Institut Curie, Paris, France. This study was approved by the Reasearch and Treatment Breast Cancer Radiation Oncology Group of the Institut Curie Hospital, Paris, France. Patients were treated between 2009 and 2015 in our Department for non metastatic breast cancer. The patients with metastatic disease, male cancers, as well as patients with implants were excluded. All patients underwent adjuvant intensity modulated radiotherapy (IMRT) with helical tomotherapy (HT) for difficult and complex volumes of irradiation, as previously reported [14]. The decision of indication of use of helical tomo therapy (HT) was taken at the weekly meeting in Department of Radiation Oncology because the impossibility to obtain satisfactory target volume coverage and dose to organs at risk sparing. During the radiation therapy all patients were followed every week, then the follow up was realised every 4 months for breast cancer patients who received systemic treatment as chemotherapy or every 6 months for the patients treated with radiotherapy +/− endocrine therapy Patients were monitored weekly during radiotherapy. Post-treatment follow-ups were conducted every 4 to 6 months, alternating between oncologists, surgeons/gynecologists, and radiation oncologists. Acute toxicities affecting the skin, gastrointestinal system, lungs, and heart were assessed retrospectively using CTCAE version 4.03.

The presented analysis is the final study of late toxicity, as well as the outcome of all population of patients. It was previewed at the beginning of registration of patients.

### 2.1. Treatment Planning Positionning CT Scans

Planning CT images and delineated volumes were transferred to the HT planning software (H iART version 2, Tomotherapy, Inc., Madison, WI, USA). Treatment plans were generated using a pitch of 0.286, an initial modulation factor of 2.5, and a collimation width of 2.5 cm. To minimize low-dose radiation to healthy tissues, two fictitious volumes were defined in the planning system, ensuring no irradiation occurred when the accelerator passed over the contralateral hemi-body or the posterior surface.

### 2.2. Target Volume Delineation

CT images were imported into the Eclipse contouring software (version 13.6; Varian Medical Systems Inc., Palo Alto, CA, USA). Clinical target volumes (CTVs) were delineated based on guidelines from the European Society of Therapeutic Radiology and Oncology (ESTRO) [16]. The primary tumor bed was contoured following previously established methods [17]. Contoured volumes were then transferred to the helical tomotherapy (HT) planning system (TomoTherapy HI-ART version 3.1.2.3; TomoTherapy Inc., Madison, WI, USA).

### 2.3. Dose Prescription

The prescribed dose for breast/chest wall and nodal areas was 50 Gy in 25 fractions (2 Gy per fraction). For cases requiring a tumor bed boost, this was delivered either sequentially (16 Gy in 8 fractions) or, more commonly, via a simultaneous integrated boost (SIB) technique. The SIB regimen delivered 52.2 Gy to the breast (1.8 Gy per fraction) and 63.8 Gy to the tumor bed (2.2 Gy per fraction) over 29 fractions. Nodal areas received a restricted dose of 50.4 Gy (1.74 Gy per fraction). The goal was to achieve homogenous coverage of 95% of the planning target volume (PTV) by the 95% isodose line.

### 2.4. Helical Tomotherapy Treatment Planning

Planning CT images and delineated volumes were transferred to the HT planning software. Treatment plans were generated using a pitch of 0.286, an initial modulation factor of 2.5, and a collimation width of 2.5 cm. To minimize low-dose radiation to healthy tissues, two fictitious volumes were defined in the planning system, ensuring no irradiation occurred when the accelerator passed over the contralateral hemi-body or the posterior surface.

### 2.5. Statistical Analysis

Statistical analyses were performed using the R programming language (version 4.0.3). Kaplan-Meier methods were applied to estimate DFS and OS curves. Multivariate Cox regression was utilized to examine the impact of disease stage and histological type on survival outcomes. DFS was defined as the interval between the end of radiotherapy and the occurrence of the first disease-related event (local, regional, or distant recurrence). Toxicity results were reported as absolute values and percentages, representing the highest grade observed during follow-up. Logistic regression analysis was conducted to identify factors associated with the development of toxicities.

## 3. Results

### 3.1. Population and Treatment

Between 2009 and 2015, a total of 194 breasts in 179 women diagnosed with non-metastatic breast cancer were treated with intensity-modulated radiotherapy (IMRT) delivered via helical tomotherapy at the Institut Curie in Paris, France. The median follow-up period following radiotherapy was 109 months (range: 6–115 months). The median age of the cohort was 53 years (range: 26–76 years), with only 25 patients (14%) being under the age of 40. Baseline patient demographics and clinical characteristics have been detailed in our prior publication [14]. Most patients (n = 164) underwent radiotherapy targeting a single breast or the chest wall, whereas 15 patients received bilateral breast or chest wall irradiation. Most of the patients were presented with lymph node positive disease (N+) (76.5%) and grade III tumors (51.4%). There were 15 patients who were presented with bilateral breast cancer (8.4%).

The patients’ and tumor characteristics are given and described in Table 1.

All patients underwent surgical intervention prior to radiotherapy. Of the treated cases, 163 breasts underwent breast-conserving surgery, while mastectomy was performed in 31 cases. Radiotherapy to the breast or chest wall was combined with lymph node irradiation in 166 breasts (85%). Among these, irradiation of all regional lymph node levels (I, II, III, IV, and internal mammary [IM] nodes) was performed in 31 cases (19%). In 145 cases (87%), radiotherapy targeted lymph node levels II, III, IV, and IM. A more limited approach, involving only level IV and IM lymph nodes, was applied in 21 cases (13%). Lymph node irradiation was omitted entirely in 28 patients.

There were only 28 patients who did not receive lymph node irradiation.

### 3.2. Survival

With a median follow-up of 10 years, we observed 9 local recurrences, 2 loco-regional recurrences, and 29 patients experienced metastatic disease. Only 18 patients are dead, of them 7 cases in relationship with breast cancer.

**Figure 1:**
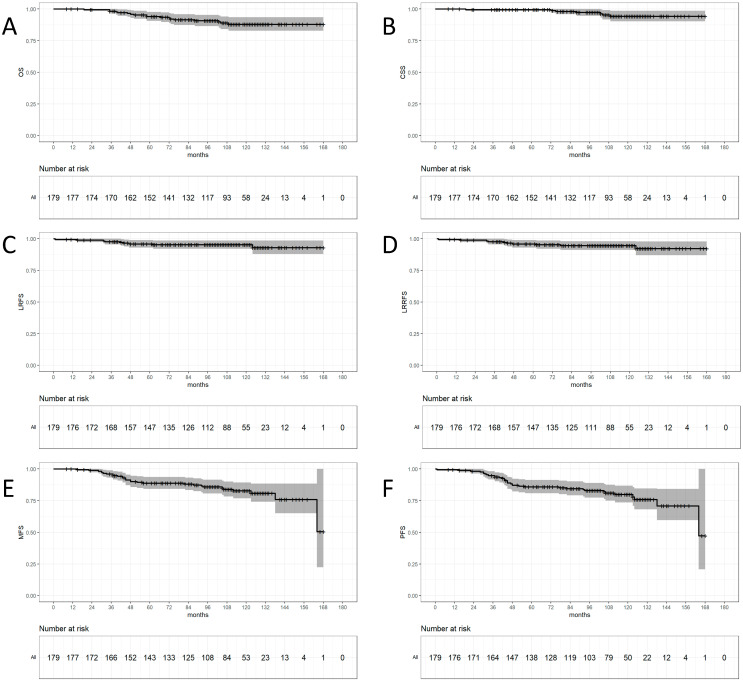
Overall survival (OS, **A**), cancer specific survival (CSS, **B**), local relapse free survival (LRFS, **C**), locoregional relapse free survival (LRRFS, **D**), metastasis-free survival (MFS, **E**), and progression free survival (PFS, **F**) of the study population.

The survival results are given and developped in Figure 1.

At 10 years, the local recurrence free survival (LRFS) was 95.3% [95%CI: 92.1–98.5], the loco-regional relapse free survival (LRRFS) was 94.5% [91.1–98.1]. The metastases free survival (MFS) was 82.9% [76.9–89.3]. The progression free survival was 79.9 [73.6–86.7]. The cancer specific survival (CSS) was 94.3%, and the overall survival (OS) was 88% [82.8–93.5].

Regarding the molecular subtype, at 10 years the progression free survival (PFS) was as following: 86.3% [74.7–99.7], 90.9% [79.6–100] and 76.7% [68.6–85.8], for triple negative cancers, HER+, and RH+/HER2—neg. tumors, respectively.

In univariate analysis, overall survival was associated with age (HR = 1.05, 95%CI: [1.01–1.10], *p* = 0.018); however no clinical or pathological variable was associated with cancer specific survival (CSS) or locoregional relapse free survival (LRRFS). The progression free survival (PFS) was associated with age (HR = 1.05, 95%CI: [1.01–1.08]), cT stage (T1: ref, T2: HR = 1.82 [0.79–4.21], T3: HR = 5.28 [1.56–9.49], T4: HR= 3.07 [1.46–19.04]). There was a non-significant trend toward reduced progression free survival in case of lymph node involvement (HR = 2.42 [0.94–6.25], *p* = 0.07).

### 3.3. Toxicity

The early toxicyty has been already reported in the previous studies and it is not a subject of this work as previosly mentioned [14,15].

At 10 years follow-up we observed 40 patients with grade I and II skin toxicities, as following: 21 cases with hyperpigmentation, 13 cases of fibrosis (of them 4 grade II), and 12 cases of teleangiectasia (of them, 2 cases of grade II). There was observed no grade III skin toxicities.

At long term, there were no cardiac, lung, thyroid, digestive radio induced toxicities.

No radiation induced sarcomas were observed in the presented series.

## 4. Discussion

To the best of our knowledge, this is one of the largest studies examining intensity modulated radiotherapy with helical tomotherapy—induced adverse effects with the longest-term outcome in breast cancer patients, as well as the outcome of patients treated for non metastatic cancers. In our cohort, intensity modulated radiotherapy with helical tomotherapy was generally well-tolerated and ensured satisfactory local disease control. Overall survival (OS) and Disease Free Survival (DFS) were very satisfactory in presented cohort of patients with high risk of recurrence, because they were mostly presented with lymph node positive (N+) cancers and grade III tumours. The final analysis with median follow-up of 10 years was programmed to exclude the long-term sequelle related to this new (in the early 2000) Tomo Therapy technique of intensity modulated radiotherapy (IMRT).

It was shown that the use of screening programs, as well as the use of modern systemic treatments and new radiation therapy techniques decresed significantly the breast cancer mortality as well as improve the local and loco regional control, as well as the quality of life [18,19,20,21,22].

Our results confirmed the already published data with low risk of skin toxicity in case of use of intensity modulated radiation therapy techniques as the Helical Tomo Therapy in comparaison with 3D radiation therapy [23,24,25]. Notably, a multicenter, double-blind randomized controlled trial conducted by Pignol et al. [23] evaluated approximately 330 breast cancer patients treated with either IMRT or conventional radiotherapy. Patients received 50 Gy in 25 fractions, followed by a 16 Gy boost. The study demonstrated a significant reduction in acute radiodermatitis in the IMRT group, with 31.2% of IMRT-treated patients experiencing this adverse effect compared to 47.8% in the standard radiotherapy group. Despite this reduction in toxicity, the study found no statistically significant difference in quality of life between the two arms.

The development of intensity modulated radiation therapy improved the treatment results because the reduction of the dose to organs at risk (OAR) as previously reported by numerous studies [23,24,25]. This reduction of the late term toxicity is usually related to better quality of life.

Other highly interesting finding is that in this population with advanced N+ tumours, bilater cancers, complex anatomy, higher doses were accepted to heart as previosly reported [14,26,27], there was not increased cardiac toxicity as it was reported by Darby et al. [4]. This fact is also in favor that the new highly performent intensity modulated radiotherapy techniques for breast and lymph node irradiation do not respect the linear model of cardiotoxicity reported for 2D and 3D techniques [4]. However, the breast cancer patients treated with 2D and 3D techniques, experienced high doses to a limited portion of the anterior heart, particularly near the posterior edge of tangential fields, where the left anterior descending coronary artery (LAD) is located.

Modern radiotherapy techniques, such as intensity-modulated radiotherapy (IMRT), including volumetric modulated arc therapy (VMAT) and helical tomotherapy (HT), allow for better sparing of the heart at the cost of exposing a larger volume to lower doses. Thus, for a similar mean hert dose (MHD), 3D techniques concentrate a high dose to a smaller heart volume, especially near the left anterior descending coronary artery (LAD), whereas intensity-modulated radiotherapy distributes a lower dose more diffusely across a larger volume. As previously reported, the side of irradiated breast (left or right) and internal mammary chain RT had no significant impact on late cardiac events in our cohort [15]. Our findings are confirming other previosly published international data [28,29,30,31,32].

There were studies repporting the increased incidence of second cancers after breast cancer radiation therapy [33]. No patients with radiation induced sarcomas were observed in presented series.

Previously, we studied also the Radio-Induced Lung Injury (RILI) and this longer follow-up report confirms that there was very low incidence of radiation induced lung injury in presented series of patients treated with tomotherapy which is in accordance with the previous reported data of limited homolateral lung V20 Gy and V30 Gy using tomo therapy [31].

Other positive message is the absence of thyroid, oesophageal, and bone (humeral head) long term toxicity.

Probably one of the futur challenges of the use of intensity modulated radiation therapy (including the tomotherapy techniques) will be the exact evaluation of the effective dose to the immune system (EDIC). This dose is mainly related to the irradiated liver volume especially in the case of patients treated to right breast or chest wall and lymph nodes. Future studies are needed to evaluate the relationship between the effective dose to the immune system (EDIC) and the patients’ outcome. The subgroup most likely to benefit from sparing the immune system includes patients with locally advanced triple-negative breast cancer (TNBC) who undergo perioperative immunotherapy and exhibit poor responses to neoadjuvant systemic therapy. These patients, characterized by a high risk of recurrence, may benefit significantly from preserving immune function to sustain effective anti-tumor responses. Establishing large-scale databases for TNBC patients receiving immunotherapy will be essential to explore this potential relationship, as has been demonstrated in esophageal and lung cancers. However, given the relatively recent adoption of immunotherapy, long-term follow-up data for this patient population remain limited. Similarly, metastatic patients with controlled distant disease undergoing locoregional treatment could theoretically benefit from approaches that minimize immune system exposure.

As previously shown, the Tomotherapy is a recognized treatment option in numerous patients with bilateral breast tumors, challenging anatomy and complex volumes [34,35].

## 5. Conclusions

With a long term median follow up of 10 years, the intensity modulated radiotherapy with helical tomo therapy appears to be safe and effective technique for complex adjuvant breast irradiation. It can be used for patients with breast cancer presented with bilateral tumours, pectus excavatum, large and complex anatomy and target volumes where the conventional 3D techniques cannot ensure an optimal dose distribution with good efficacy and tolerance. The decreasing of long term adverse effects related to radiation therapy could be translated by improvement of the patients’ quality of life.

## Figures and Tables

**Table 1:** Patients’ and tumour characteristics.

Patient Characteristics	Number (n = 179)	%
**Age, median [range] (y.)**	53 [25–76]	
**Side**		
Left	80	44.69
Right	84	46.93
Bilateral	15	8.38
**T stage**		
Tis	5	2.79
T1-T2	158	88.27
T3-T4	30	16.76
Tx	1	0.56
**N stage**		
N0	57	31.84
N+	137	76.54
**Grade**		
I	17	9.50
II	78	43.58
III	92	51.40
NA	7	3.91
**Histological type**		
HR+	130	72.63
HER2 positive	25	13.97
TNBC	33	18.44
NA	6	3.35
**Radiotherapy**		
Fraction number, median [range]	29 [13–34]	
Breast/Chest wall dose, median [range], Gy	52.2 [40–52.2]	
Chest wall irradiation	31	15.98
Whole breast irradiation	163	84.02
*Inc. Tumor bed boost*	*152*	*93.25*
Regional node irradiation	165	85.05
*Inc. Internal mammary chain*	*31*	*18.79*

HR: hormone receptor. TNBC: triple negative breast cancer. NA: non assessable.

## Data Availability

In case of questions, the complementary data could be available.
